# Arabinoxylans Release from Brewers’ Spent Grain Using Extrusion and Solid-State Fermentation with *Fusarium oxysporum* and the Antioxidant Capacity of the Extracts

**DOI:** 10.3390/foods11101415

**Published:** 2022-05-13

**Authors:** Joel G. Cervantes-Ramirez, Francisco Vasquez-Lara, Alberto Sanchez-Estrada, Rosalba Troncoso-Rojas, Erick Heredia-Olea, Alma R. Islas-Rubio

**Affiliations:** 1 Coordinación de Tecnología de Alimentos de Origen Vegetal, Centro de Investigación en Alimentación y Desarrollo, A.C., Carretera Gustavo E. Astiazarán Rosas # 46, Colonia La Victoria, Hermosillo 83304, Mexico; joel.cervantes.mc19@estudiantes.ciad.mx (J.G.C.-R.); fvas@ciad.mx (F.V.-L.); aestrada@ciad.mx (A.S.-E.); rtroncoso@ciad.mx (R.T.-R.); 2 Centro de Biotecnología FEMSA, Instituto Tecnológico y de Estudios Superiores de Monterrey, Ave. Eugenio Garza Sada No. 2501, Monterrey 64849, Mexico; erickho@tec.mx

**Keywords:** brewers’ spent grain, arabinoxylans, extrusion, solid-state fermentation, *Fusarium oxysporum*, antioxidant capacity

## Abstract

Brewers’ spent grain (BSG) is the most abundant byproduct generated from the beer-brewing process. BSG is a material rich in hemicellulose, composed of arabinoxylans (AX). However, the high crosslinking of this material causes low availability of AX, for which it is necessary to apply different treatments. The objective of this research is to increase the release of arabinoxylans through solid-state fermentation with *Fusarium oxysporum* f. sp. *lycopersici* using extruded brewery spent grain. First, the BSG is subjected to two types of physical treatments: extrusion at 20% moisture, 200 rpm and 50 °C (BSGe), and blade milling (BSGm). The chemical composition is determined for each sample (BSG, BSGe and BSGm). Subsequently, the solid-state fermentation process (SSF) is carried out on each sample. The fermentation kinetics at 30 °C are monitored for 7 days. Once the SSF concludes, AX are extracted, and the purity of AX is determined by the phloroglucinol colorimetric assay. Finally, the total phenolic compounds, phenolic acids and antioxidant capacity by DPPH are quantified. No significant differences (*p* ≥ 0.05) in the protein, lipid, ash or total dietary fiber contents are found among the samples. No significant difference (*p* ≥ 0.05) in the content of soluble fiber is found, although BSGe and BSGm have higher values than BSG. On the other hand, the yields of soluble AX exhibit significant differences (*p* ≤ 0.05) among nonfermented samples (BSG, 0.03%; BSGm, 0.53%; BSGe, 0.70%) and with SSF (BSG, 2.95%; BSGm, 6.24%; and BSGe, 9.58%). In addition, the contents of free phenolic compounds and free phenolic acids and the percent inhibition of free extracts by 2,2-diphenyl-1-picrylhydrazyl (DPPH) differ significantly (*p* ≤ 0.05) between samples subjected to SSF and nonfermented samples. Therefore, extrusion and SSF treatment increase AX release from BSG as well as the antioxidant capacity of the extracts.

## 1. Introduction

Brewers’ spent grain (BSG) is the main residue generated during the mashing stage of the beer production process and represents 85% of the waste obtained in the beer industry. Annually, approximately 39 million tons of BSG are produced worldwide [1]. The high moisture (70%) and polysaccharide (40–50% d.w.) contents of BSG make it susceptible to microbial growth and deterioration after a few days of storage at ambient temperature [2]. BSG can be used as a supplement for animal feed or—in some instances—as food for human consumption, or it is disposed of in fields. An overview of the utilization of brewery byproducts as generated by British craft breweries is reported [3]. However, interest in reductions in industrial waste and its reutilization in biotechnological applications to obtain high value added products has made BSG the subject of studies.

The chemical composition of BSG varies with the type of beer and processing conditions; nevertheless, it is made up of mostly lignocellulosic material (30–50%), of which hemicellulose represents between 20 and 25% [4]. Hemicellulose is composed of heteropolymers; however, cereals contain greater proportions of pentosans, also known as arabinoxylans [2]. Bonifácio-Lopes et al. [5] reported a composition of BSG of 25% hemicellulose, 20% cellulose, 18% lignin, 22% protein and 15% lipids and other compounds.

Arabinoxylans are made up of linear chains of *β*-(1→4)-xylopyranose, and the *α*-l-arabinofuranose units are attached to the main xylose chain at positions two and/or three. Arabinose molecules can be esterified with hydroxycinnamic acids, monomeric or dimeric ferulic acid and *p*-coumaric acid [6]. AX has been applied as an emulsifying agent, film-forming agent, gelling agent, antioxidant and prebiotic [7]. However, the high crosslinking of lignocellulosic material results in the low availability of AX and ferulic acid [8].

In order to access the high amounts of bioactive compounds present in BSG, various treatments have been used to increase their availability using biological, physical, enzymatic and chemical methods [9,10]. The latter have been the most widely used to obtain AX; despite this, the use of chemical agents changes functional properties and tends to produce fractions of high molecular weight (100–200 kDa) and to release ferulic acid, in addition to generating hazardous waste to the environment. On the other hand, enzyme treatment releases AX with greater specificity, but commercial success is limited by the high price of commercial enzymes [11].

Physical methods have the objective of fragmenting lignocellulosic material to increase accessibility to hydrolysis. Among the physical treatments for BSG are the liquid-hot water process [5], microwave irradiation [12], ultrasound application [13] and extrusion [14]. During extrusion, the raw material is exposed to conditions of high temperature, pressure, speed and shear, which leads to physical and chemical changes [15]. The extrusion process has recently been used to increase solubility and decrease the molecular weight of AX from food sources and agro-industrial waste [9]. Finally, biological methods use microorganisms to degrade cell walls by secreting enzymes to hydrolyze polymers into monomers, and they carry out the growth of microorganisms using submerged fermentation (SmF) and solid-state fermentation (SSF) [16].

Xiros and Christakopoulos [17] modify BSG using chemical agents and a fungal extract of *Fusarium oxysporum* by means of SmF, and the production of xylanases and feruloyl esterases can be observed. On the other hand, the SSF process from filamentous extracts has been studied for the production of enzymes (xylanases and feruloyl esterases), which are the same enzymes that are used under the enzymatic method to obtain AX. This procedure enjoys several technological and operational advantages over SmF [18], since there is a greater production of enzymes. The extrusion of BSG in combination with SSF using *Fusarium oxysporum* f. sp. *lycopersici* can be a good alternative for releasing AX and ferulic acid from BSG. Therefore, this research focuses on increasing the content of AX from fermentation in the solid state with a strain of *Fusarium oxysporum* f. sp. *lycopersici* using BSG treated by extrusion as a substrate.

## 2. Materials and Methods

### 2.1. Material

BSG was procured from Heineken México (Monterrey, México). The wet BSG was transported to the Tecnológico de Monterrey campus, Monterrey, and was dried in an oven (Zanussi, Pordenone, Italy at 45–50 °C for 48 h. The fungus *Fusarium oxysporum* f. sp. *lycopersici* (*Fol*) was procured from Laboratorio de Biotecnología Vegetal y Postcosecha (CIAD, Hermosillo, México). The fungus was isolated from tomato fruit (*Solanum lycopersicum*) and was identified according to the morphological and microscopic characteristics reported [19,20].

### 2.2. Milling Process

The dry BSG was milled (BSGm) using a blade mill (Wiley Mill^®^, Swedesboro, NJ, USA) equipped with a 2 mm screen. The sample was packed in plastic bags (Ziploc^®^, Racine, WI, USA) and stored at room temperature.

### 2.3. Extrusion Process

A twin-screw corotating extruder (BTSM-30, Bühler AG, Uzwil, Switzerland) with a barrel composed of 5 zones and 2 independent feeders for the solid raw material and water was used. The temperature of the fifth zone of the barrel was controlled by a heat exchanger device (Tool Temp, Bühler AG, Uzwil, Switzerland). The total length and outer diameter of the screws were 800 mm and 30 mm, respectively, and the L/D ratio was 20. A die with a single 4 mm hole was used. The screw configuration consisted of three different sections: an inlet/conveying element section (for the introduction and transport of the dry feedstock and water), a mixing element section and a final work element section consisting of kneading and reverse elements. The dry unmilled BSG was introduced into the hopper to be extruded (BSGe). The screw speed, temperature, and moisture were fixed at 200 rpm, 50 °C, and 20 %, respectively, and these are the parameters established by [14]. The BSGe was dried in an oven (Zanussi, Pordenone, Italy) at 50–60 °C for 1 h and was stored in plastic bags for subsequent analyses.

### 2.4. Chemical Characterization

Only the structural carbohydrate assay was performed on BSG; the nonstructural material was removed from the BSG to prevent any interference. More specifically, a two-step extraction process was used to remove water and ethanol solubles according to the methods recommended by the National Renewable Energy Laboratory (NREL) [21]. Then, the insoluble fibers were hydrolyzed and filtered for structural sugar quantification with high-performance liquid chromatography (HPLC), as described by [22]. Next, moisture, ash, total dietary fiber, protein and lipid contents were determined for each sample (BSG, BSGm and BSGe) using the approved methods 44-15.02, 08-01.01, 32-05.01, 46-13.01 and 30-20.01 of the American Association of Cereal Chemists [23], respectively.

### 2.5. Inoculum

A strain of the fungus *Fusarium oxysporum* f. sp. *lycopersici*, isolated from tomato fruit (collection of the Laboratorio de Biotecnología Vegetal y Poscosecha, CIAD, Hermosillo, México), was used. The fungus was initially grown on potato dextrose agar (PDA) (BD Bioxon^®^, Guadalajara, México) and was incubated (Shel Lab^®^ SMI2, Cornelius, OR, USA) at 30 °C for 10 days. For inoculum preparation, 15 mL sterile deionized water with 100 µL Tween 80 was added to the fungus colony surface. The colony surface was scraped, and the spore suspension was adjusted to a concentration of 1.3 × 10^7^ spores/mL using a Neubauer chamber (Bright-Line, Buffalo, NY, USA) [24]. Aliquots of spore suspension (5 mL) were taken and added to 100 mL of the mineral medium, as described by [25]. An amount of 20 g/L BSG was added, and the pH was adjusted to 6.0 by the addition of hydrochloric acid. The flasks were incubated at 30 °C for 3 days in an orbital shaker (250 rpm) for mycelium production.

### 2.6. Solid-State Fermentation

The SSF assay was performed according to the method described by [25], with some modifications. SSF was carried out in 250 mL Erlenmeyer flasks containing 50 g of carbon source (BSG, BSGm or BSGe), and each sample was moistened with 10 mL of mineral medium until a water activity (Aw) of 0.9 was reached. Aw was measured by an AquaLab instrument (4Te, METER Group, Inc., Pullman, WA, USA). Prior to sterilization, the initial pH of the medium was adjusted to 6.0 by the addition of hydrochloric acid. The medium was sterilized at 110 °C for 15 min (Yamato SE-510, Yamato Scientific America, Inc., Santa Clara, CA, USA). The production culture medium was inoculated with 10 mL of 72 h-old culture (prepared as described above), and this process was carried out inside a laminar flow hood (Labconco, Kansas City, MI, USA). The flasks were incubated at 30 °C for 7 days. Three flasks were removed in each time interval (24, 48, 72, 96, 120, 144 and 168 h) and sterilized at 121 °C and 15 psi for 15 min to stop the fermentation process, after which the extraction of soluble AX was performed according to the established treatment, as described in the next section.

### 2.7. Extraction and Purification of Water-Extractable Arabinoxylans

AX were extracted and purified using the method described by [9], with some modifications. In summary, 25 g of each sample was added to a centrifuge tube with a capacity of 250 mL, and 225 mL of water was added. The tubes were incubated in a shaking water bath (Hot Shaker, Bellco Glass, Inc., Vineland, NJ, USA) for 2 h at 40 °C. Following centrifugation at 6000× *g* for 40 min at 10 °C (Hermle Labortechnik GmbH, Wehingen, Germany), supernatants were adjusted to a pH of 7 using 1 M NaOH or 1 M HCl before incubating with 183.75 μL thermostable α-amylase (340 Units/mg) (Thermozyme^®^ L340, ENMEX, Tlalnepantla, México) in a shaking water bath (Mirak, Thermolyne, Corp., Dubuque, IA, USA) at 91 °C for 60 min. Samples were then cooled at room temperature before removing the protein, with the addition of 104.16 μL Alcalase (2.4 Units/mg) (Novozymes, Bagsvaerd, Denmark) at 50 °C for 14 h. The proteinase was then deactivated by placing samples in a boiling water bath for 10 min. Samples were allowed to cool at room temperature and were centrifuged at 4600× *g* for 20 min. Ethanol (70:30 *v/v* in distilled water) (DEQ, Monterrey, México) was added to each supernatant at 4 °C, which was kept overnight. Precipitates were recovered by centrifugation at 4600× *g* for 20 min at 10 °C. The supernatants were discarded, and the residue was retained. The residue was weighed before washing and vortexing twice with 20 mL absolute ethanol (minimum 99%) (DEQ, Monterrey, México). Finally, 20 mL acetone (DEQ, Monterrey, México) was added, and the samples were vortexed (Vortex Mixer, Labnet, Edison, NJ, USA) for one minute followed by centrifugation at 4600× *g* for 20 min at 10 °C. The final precipitates were dried for 48 h at 45 °C in a drying oven (Fisher Scientific, Pittsburgh, PA, USA). Once dry, the extracts were weighed and stored in plastic bags at 21 °C until further analysis.

### 2.8. Determination of Arabinoxylan Content

A colorimetric assay was employed to determine the content of pentosans (arabinose and xylose) following the method described by [26]. In summary, D-xylose (Sigma, St. Louis, MO, USA) at concentrations of 0.05, 0.15, 0.25, 0.5, 0.75 and 1 mg/mL was used to construct the calibration curve. The colorimetric reagent was prepared by mixing 5 mL of phloroglucinol in absolute ethanol (20% *w*/*v*) with 110 mL of glacial acetic acid (DEQ, Monterrey, México), 2 mL of hydrochloric acid and 1 mL of glucose solution in water (1.75% *w*/*v*) (Sigma, St. Louis, MO, USA). Triplicate 2 mL aliquots of each standard dilution were mixed with 10 mL of the colorimetric reagent. Immediately, the tubes containing the mixtures were placed in a boiling water bath (Mirak, Thermolyne, Dubuque, IA, USA) for 25 min. The samples were removed, cooled in an ice bath for 1 min and were immediately placed for 1 min in a water bath adjusted to room temperature. The tubes were removed, laid horizontally, and covered with aluminum foil. After 10 min, the absorbance values of the samples were read at 552 and 510 nm using a Genesys 10S UV-Vis spectrophotometer (Thermo Scientific, Milford, MA, USA). The absorbance reading at 510 nm was subtracted from that of the reading at 552 nm to remove the influence of hexoses. A portion (5 mg) of each experimental sample (BSG, BSGm, BSGe) was added to a separate solution containing 2 mL of water and 10 mL of the colorimetric reagent. The AX content in each sample was determined by interpolation with the equation derived from the standard xylose curve. The total pentosan content in each sample was determined in triplicate, and the values are reported as percentages.

### 2.9. Extraction of Free Phenolic Compounds

Free phenolic compounds were extracted according to the method of [27], with some modifications. An amount of 2 grams of each sample was used and defatted two times with 20 mL hexane (DEQ, Monterrey, México) to remove lipids by shaking with a wrist-action shaker bath (Labline Instruments, Inc., Dubuque, IA, USA) for 15 min under dark conditions at room temperature followed by centrifugation at 10,000 rpm (Thermo Fisher Scientific, Waltham, MA, USA) and 4 °C for 10 min. The lipid-free residue was extracted three times with 80% methanol (20 mL) (DEQ, Monterrey, México). Each time, the mixture was shaken on a wrist-action shaker for 1 h in the dark at room temperature. The mixture was then centrifuged at 10,000 rpm at 4 °C for 10 min. The combined supernatants were filtered and evaporated to dryness under vacuum at 38 °C with a rotary vacuum evaporator (LABORATA 4010-digital, Heidolph, Germany). The dried extracts were redissolved in 50% methanol (5 mL) and used as crude-free phenolic extracts. The extracts were stored at −20 °C (Intelligent Freezer 32, Bosch, Guadalajara, México) until the TPC, DPPH and HPLC analyses. The extractions and analyses were performed in duplicate.

### 2.10. Extraction of Bound Phenolic Compounds

Bound phenolic compounds were extracted according to the method described by [27], with modifications. The residue collected after methanol extraction was hydrolyzed with 40 mL of 4 M NaOH (DEQ, Monterrey, México) at room temperature for 2 h. The mixture was shaken using a wrist-action shaker (Labline Instruments, Inc., Dubuque, IA, USA) in the dark at room temperature. The hydrolyzed mixture was adjusted to a pH of 1.5–2.0 with 6 M HCl (Hanna Instruments, Padua, Italy). After centrifugation at 10,000 rpm for 10 min at 4 °C, the supernatant was extracted three times with 50 mL ethyl acetate (total 150 mL). The combined ethyl acetate fractions were evaporated to dryness under vacuum at 35 °C with a rotary vacuum evaporator (LABORATA 4010-digital, Heidolph, Germany). The dried extracts were redissolved in 50% methanol (5 mL) and were used as crude-bound phenolic extracts. The extracts were stored at −20 °C (Intelligent Freezer 32, Bosch, Guadalajara, México) until the TPC, DPPH and HPLC analyses. The extractions and analyses were performed in duplicate.

### 2.11. Determination of Total Phenolic Content (TPC)

Total phenolic content was determined using the Folin–Ciocalteu colorimetric method described by [27], with some modifications. Briefly, instead of 200 μL of appropriately diluted crude extract or standard solution, 100 μL was added to the 1.5 mL of 10-fold freshly diluted Folin–Ciocalteu reagent. The rest of the procedure described by [27] was followed, and the TPC was reported as milligrams of gallic acid equivalent per 100 g of sample (mg GAE/100 g dry weight).

### 2.12. Determination of Phenolic Acids Using HPLC

HPLC assays were conducted as described by [27], with some modifications. Chromatographic separation was carried out on an RP-HPLC (Agilent Technologies, Walnut Creek, CA, USA) equipped with a photodiode array detector. The analytical column was a C18 110A column, 300 mm × 4.6 mm, with a particle size of 5 μm (Beckman Coulter, Brea, CA, USA). The mobile phase consisted of A (100% acetonitrile) and B (0.1% formic acid in water). The analytes were eluted using an isocratic separation with 8% A and 92% B from the injection time until 35 min. The conditions were set as follows: 24 °C column temperature, 1 mL/min flow rate and 10 μL injection volume. The phenolic acids were detected at a wavelength of 320 nm and were identified by comparing retention times with those of their respective standards. The contents of phenolic acids were quantified using external calibration curves.

### 2.13. Determination of DPPH Radical Scavenging Activity

The DPPH radical scavenging activity assay was carried out according to [27], without modifications. The scavenging activity (%) of the DPPH radical of both the sample and the standard (Trolox) was calculated as reported in [27], and the DPPH values are expressed as micromoles of Trolox equivalent per gram of sample (μmol TE/g dry weight).

### 2.14. Experimental Design and Statistical Analysis

Table 1 shows the experimental design used in the present work. A 2^2^ complete factorial design was applied to evaluate the effects of the factors (A) extrusion process (BSG without extrusion and BSG extruded) and (B) solid-state fermentation with *Fol* (BSG unfermented and BSG fermented) on the percentage of arabinoxylan release from BSG. All analyses were performed in triplicate, and the results are expressed as the mean ± standard deviation. Statistical analyses were conducted by one-way ANOVA, and differences among means were compared by Tukey tests with a level of significance of *p* ≤ 0.05. All statistical analyses were performed by using NCSS (statistical analysis software, Kaysville, UT, USA).

## 3. Results and Discussion

### 3.1. Chemical Characterization

The structural carbohydrates from the BSG are shown in Table 2. The contents of water-soluble extractives and ethanol extractives were 10.9% and 13.2%, respectively. This indicates that BSG contained 24% nonstructural material, and these extracts comprised the nonstructural components that could have possibly interfered with the characterization of the raw material [22].

On the other hand, the value for lignin is 18.9%, that for hemicellulose is 26.4% and that for cellulose is 13.8%, and these values are within the range reported previously [17,28]. Hemicellulose is the main BSG constituent, primarily consisting of AX. However, BSG contains a high lignin content, even higher than those in sugarcane bagasse, rice straw and barley straw (between 15–18%), which influences the release of AX due to steric hindrance. Table 3 shows the chemical composition of BSG, BSGm and BSGe. The results show that there is no significant difference (*p* ≥ 0.05) in the contents of ash, lipids, protein or total dietary fiber among the samples, and these values are within the range reported previously [2,29,30,31].

The insoluble fiber contents of BSG, BSGm and BSGe are 61.6%, 61.8% and 60.7%, respectively, and the amounts of soluble fiber in BSG, BSGm and BSGe are 0.2%, 2.0% and 2.2%, respectively. Therefore, there is a significant difference (*p* ≤ 0.05) in soluble and insoluble fiber in BSG between the samples treated by physical processes (BSGm and BSGe). The increase in soluble fiber content was due to the cell walls in BSG, which are constructed by crosslinking, making it difficult for the material to be soluble in an aqueous medium [32]. Therefore, the physical process breaks some crosslinking. The extrusion and milling process modified the soluble fiber, and when comparing the soluble fiber values, it was found that there were increases of 10 times more in BSGm and 11 times more in BSGe compared to values in BSG (Table 3). These results are of great interest due to the nutritional and functional benefits that soluble fiber provides to human health [33].

Vasanthan et al. [34] evaluated the effects of extrusion conditions on total, soluble and insoluble fibers in barley flour and concluded that soluble fiber content is reduced due to extrusion cooking. On the other hand, Espinosa-Ramírez et al. [35] compared different whole grains, pseudocereals and legumes to produce techno-functional flours using extrusion and reported that in four out of nine samples, soluble dietary fiber was higher than in samples of unextruded flour. The extrusion process has been characterized as disadvantageous because it can decrease dietary fiber. However, this reduction has been associated with the release of oligosaccharides from insoluble fiber and soluble fiber, making them digestible during the test and thus avoiding their quantification [36].

### 3.2. Soluble Arabinoxylan Content in BSG Extracts

**Figure 1:**
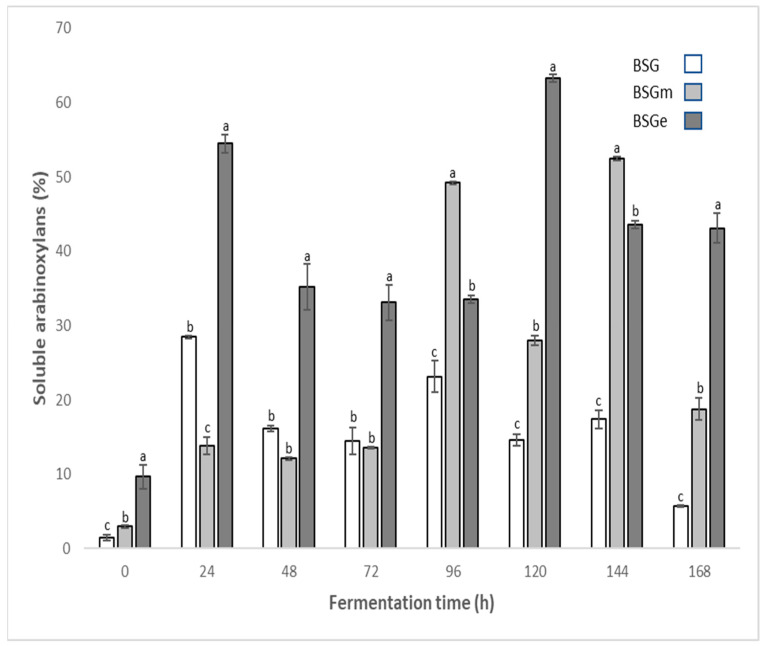
Content of soluble arabinoxylans in extracts of brewers’ spent grain (BSG), BSG milled (BSGm), and BSG extruded (BSGe), and the fermented counterparts by *Fusarium oxysporum* up to 168 h. Significant differences for each fermentation time are shown by the letters a, b, and c.

The results obtained for the content of soluble arabinoxylans in unfermented BSG, BSGm and BSGe as well as in the corresponding samples fermented by *Fusarium oxysporum* for 24 h to 168 h are reported in Figure 1. The results show that the samples not subjected to the SSF process showed lower values of soluble AX. The highest levels of soluble AX occurred during the SSF process, and the highest value (63.28%) was for BSGe (*p* ≤ 0.05) compared to values of 28.47% and 52.45% for BSG and BSGm, respectively. During the SSF process, the content of soluble AX in extracts increased in each sample. This may be due to the xylanases enzymes produced by the fungus, which are responsible for hydrolyzing xylan and converting it to xylose and xylooligosaccharides because they break glycosidic bonds either by an inversion or a retention mechanism in which the anomeric carbon either inverts or retains its stereochemical configuration [37]. At the end of the SSF process, soluble Ax contents in extracts declined due to the SSF process, because the microorganism could use these polysaccharides during metabolic processes and growth or because the substrate could be depleted. Sanchez [16] classified *Fusarium oxysporum* as a type of brown rot fungus, which shows greater degradation of cellulose and hemicellulose through the production of these enzymes (cellulases, xylanases) to convert polymers into oligomers or monomers for use by the fungus.

The type of enzyme is a determining factor in the increase in AX values. In this case, xylanases are classified according to their physicochemical properties; however, the most significant xylanases are GH10 and GH11. GH10 xylanases have been found to be more active on soluble substrates, whereas GH11 prefers insoluble polymeric xylanes, which release more soluble AX in an aqueous medium. Various authors have reported greater production of GH10-type [38,39,40] than GH11 [41,42] xylanases by *Fusarium oxysporum*, which may be of interest for investigating the type of xylanases generated during fungal metabolism.

Fermentation is a dynamic process. In general, there is a positive effect of physical treatment on the soluble arabinoxylans through the fermentation process. High variation in the AXs over time during fermentation may be due to the action of xylanases and other enzymes (feruloyl esterases, peptidases, carbohydrases, etc.) secreted by the fungus [43]. It has been reported that the hydrolysis of cell wall polysaccharides in BSG increases protein solubilization [44]. Most likely, new protein–polysaccharide, phenolic acid–polysaccharide and phenolic acid–protein interactions can take place; therefore, a decrease in phenolic acids can occur. Different enzymes secreted by the fungus can differ in secretion time and their activities. This hypothesis needs to be confirmed by further studies.

### 3.3. Yield of Soluble Arabinoxylans from BSG

**Figure 2:**
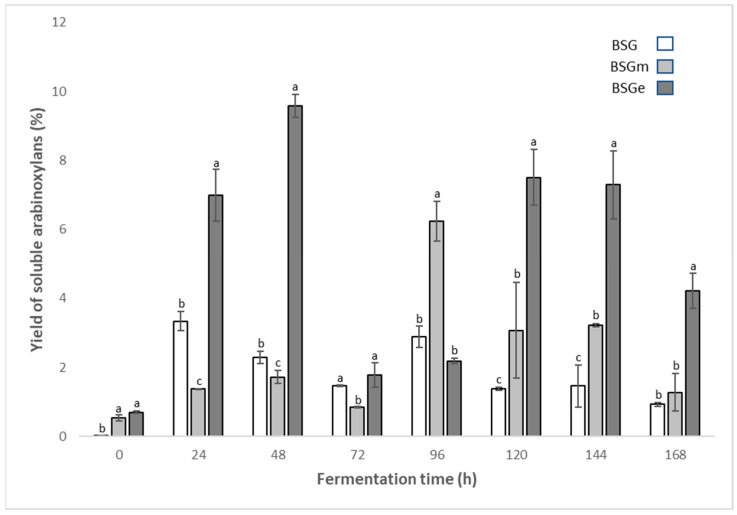
Yields of soluble arabinoxylans in unfermented samples of brewers’ spent grain (BSG), BSG milled (BSGm), BSG extruded (BSGe) and the fermented counterparts by *Fusarium oxysporum* up to 168 h. Significant differences for each fermentation time are shown by the letters a, b, and c.

The yields of soluble arabinoxylans from BSG, BSGm and BSGe and from fermented BSG, BSGm and BSGe by *Fusarium oxysporum* from 24 h to 168 h are shown in Figure 2. The yield of soluble AX from BSGm was 0.53%, and that from BSGe was 0.70%. No significant difference between these values (*p* ≥ 0.05) was found. Although the extrusion process did not show a significant difference with respect to the milling process, the main advantages of the extrusion process include low energy consumption and the continuity of the process. On the other hand, there was a significant difference (*p* ≤ 0.05) in BSG, which generated a yield of soluble AX of 0.03%, which translates into an increase of 17 times more in BSGm and 23 times more in BSGe. This increase in soluble AX is due to physical processes, because these processes fragment the crosslinking that hinders solubility in aqueous media [32].

The SSF process produced higher yields, with BSGe displaying the highest value, which was 9.58%, significantly different (*p* ≤ 0.05) from values for BSG (2.95%) and BSGm (6.24%). This indicates that 3 times more AX are obtained when using fermented BSGe than when using fermented BSG, and 1.5 times more AX are produced when using fermented BSGe than when using fermented BSGm. On the other hand, the yield obtained from fermented BSGe when compared with samples not subjected to the SSF process had a yield of AX of 319 times more than that of BSG, 18 times more than that of BSGm and 13 times more than that of BSGe. Therefore, there is a significant interaction effect (*p* ≤ 0.05) of the type of physical treatment (extrusion or milling) and biological treatment (SSF) on the yield of soluble AX present in BSG.

Different researchers have reported the yields of soluble AX extracted from BSG. Coelho et al. [45] used BSGm provided by a company and extracted AX with water (20 °C) and hot water (80 °C), obtaining yields of 0.5%. Compared with the results obtained in our work, the values are similar to those for the samples not subjected to SSF. On the other hand, Reis et al. [13] used BSGm provided by a microdistillery with the objective of increasing the extraction of soluble AX through ultrasound application and autoclaving, obtaining a yield of 3.1%. In comparison with the results obtained in our work, the values are similar to those for fermented BSGe. Severini et al. [46] reported a yield of 10.3% soluble arabinoxylans in BSG by enzymatic treatment, which is close to that obtained in our work (9.58%). Coelho et al. [45] and Reis et al. [13] obtained yields of 20.8 and 20.7%, respectively; however, these research groups used chemical treatments for the extraction of AX. These differences may be due to the use of chemical agents, because the use of chemical agents increases the solubility of hemicellulose, which makes hemicellulose more accessible [47].

During the SSF process, the maximum release of water-soluble AX occurred at 48 h in BSGe, whereas in BSGm and BSG, it occurred at 96 h. This result is important for optimizing SSF time and thereby for reducing processing costs. Only the samples fermented for these two periods were taken for the quantification of total phenolic compounds, phenolic acids and antioxidant capacity, which are described below.

### 3.4. Total Phenolic Compounds

The TPC in BSGe was 2.28 mg GAE/g, differing significantly (*p* ≤ 0.05) from values for BSG and BSGm, which were 1.70 mg GAE/g and 1.26 mg GAE/g, respectively. This increase may be due to the mechanical operation process, because the extruder uses high pressure, temperature and shear, which degrade lignocellulosic material, and this may increase bound and free phenols [46]. On the other hand, Ti et al. [48] evaluated the effect of extrusion on phytochemical profiles in milled rice fractions and concluded that the use of the extruder increased TPC in rice bran. Therefore, the use of extrusion may have caused the increase in the TPC in BSG.

The concentration of free phenolic compounds in BSG was 0.67 mg GAE/g, significantly different (*p* ≤ 0.05) from those of BSGm and BSGe, with values of 0.58 (mg GAE/g) and 0.59 (mg GAE/g), respectively. However, the proportion of free phenolic compounds was higher in BSGm (45.74%) than in BSG and BSGe, which showed proportions of 39.40% and 25.71%, respectively. On the other hand, it has been reported that free phenolic compounds in BSG with the use of methanol as a solvent for extraction range from 0.5 mg GAE/g to 6.46 mg GAE/g [49,50]; therefore, the results obtained in our study are within the reported range.

TPC and free phenolic compounds were higher in the SSF process than in the samples that were not subjected to SSF, and this is apparent during the first 48 h of fermentation. This change is due to the enzymes (e.g., xylanases, feruloyl esterases) that the fungus produces during SSF. These enzymes break down the cell walls [16], and the breaking of bonds between cellulose or hemicellulose and carboxyl groups on phenolic acids causes the release of the bound phenolics. In addition, SSF has been reported to increase the release of phenolic compounds in cereal byproducts (e.g., wheat bran, corn nejayote, and brewery mastic) [51,52,53]. However, the samples fermented for 96 h showed low values of TPC and bound phenolic compounds, perhaps because the fungi produced extracellular enzymes, which are involved in the degradation and conversion of the phenolic compounds present in lignocellulose. These enzymes (e.g., laccase, heme-thiolate peroxidase, feruloyl esterase, tannase) hydrolyze large phenolic compounds to smaller subunits that can then be metabolized by fungal cells [54]. However, no information has been found on the transport of phenolic compounds from the growth medium to fungal cells [55].

### 3.5. Phenolic Acids

**Figure 3:**
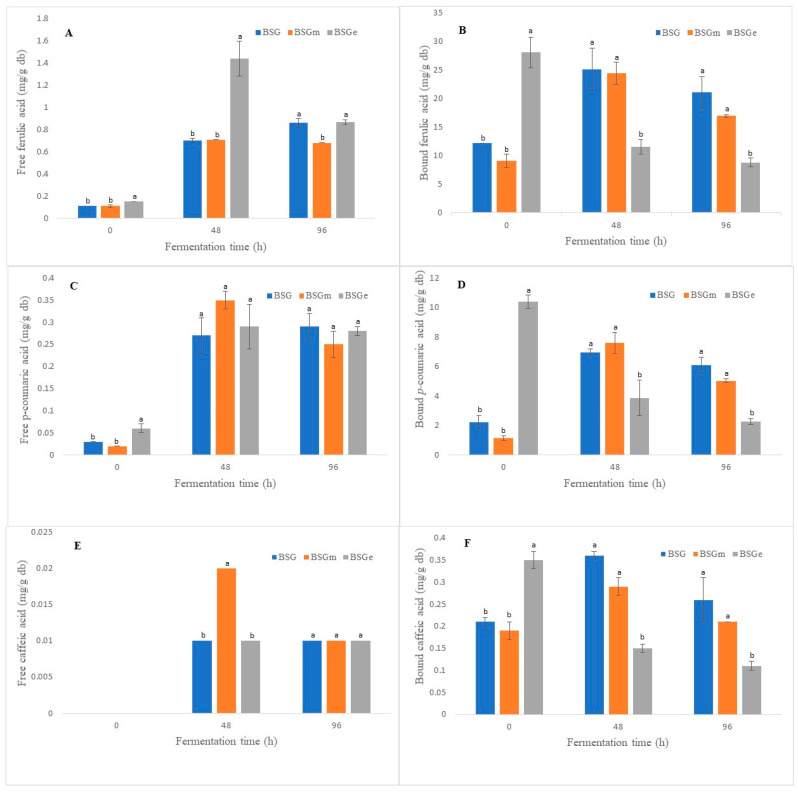
Free and bound phenolic acids from unfermented samples (brewers’ spent grain, BSG; BSG milled, BSGm; BSG extruded, BSGe) and the fermented counterparts by *Fusarium oxysporum* after 48 h and 96 h. Subfigures correspond to free ferulic acid (**A**), bound ferulic acid (**B**), free *p*-coumaric acid (**C**), bound *p*-coumaric acid (**D**), free caffeic acid (**E**), and bound caffeic acid (**F**). Significant differences for each fermentation time are shown by the letters a, and b.

The free and bound phenolic acids in the nonfermented samples and the fermented counterparts are shown in Figure 3. Chromatographic analysis revealed the presence of three phenolic acids in the samples: ferulic, *p*-coumaric and caffeic acids. Ferulic acid was the most abundant in the samples that were not fermented, with values of 12.28 mg/g in BSG, 9.21 mg/g in BSGm and 28.19 mg/g in BSGe for bound ferulic acid (Figure 3B) and values lower than 0.2 mg/g for free ferulic acid (Figure 3A). It has been reported that ferulic acid is the hydroxycinnamic acid with the highest presence in BSG [56], which is consistent with our results.

The bound *p*-coumaric acid contents of nonfermented samples (0 h) were 2.27 mg/g BSG, 1.17 mg/g BSGm and 10.45 mg/g BSGe (Figure 3D), whereas the free *p*-coumaric contents in these samples were below 0.1 mg/g (Figure 3C). Various authors have reported *p*-coumaric acid as the phenolic acid with the second highest concentration in BSG; however, the values obtained in this study are higher than those reported (which range from 0.6 mg/g to 1.1 mg/g) [57,58]. On the other hand, the bound caffeic acid contents in nonfermented samples were 0.21 mg/g for BSG, 0.19 mg/g for BSGm and 0.35 mg/g for BSGe (Figure 3F), which are higher concentrations than those reported by [56] (0.1 mg/g). These differences may be due to the type of malt used for brewing, the type of adjunct used, and the extraction technique. Extraction technique affects the content of phenolic compounds, as do the type of solvent used, the reaction or extraction time, temperature and/or the physical treatments used [50].

On the other hand, free phenolic acid contents were higher in the samples subjected to SSF (Figure 3A,C,D). However, BSGe differed significantly (*p* ≤ 0.05) in free ferulic acid from BSG and BSGm, and the results showed that at 48 h of fermentation, 1.44 mg/g of free ferulic acid was obtained, whereas at 96 h, it decreased to 0.87 mg/g (Figure 3A). This change can be explained by the use of hydroxycinnamic acids by the fungus for metabolism [54] or by the depletion of the substrate. Further studies are needed to test this hypothesis. The quantification of soluble AX is of great importance for confirming the production of enzymes during the SSF process, which releases AX and produces a greater amount of free ferulic acid.

### 3.6. Antioxidant Capacity by DPPH

Values for the antioxidant capacity of free and bound phenolic compounds in the extracts of BSG, BSGm and BSGe unfermented and fermented by *Fol* after 48 h and 96 h are shown in Table 4. The DPPH assay is based on the capacity of the DPPH radical to react with hydrogen donors, i.e., the phenols present in each sample. Verni et al. [59] evaluated free phenolics in BSG extracts and reported 42.5% inhibition of DPPH, whereas Reis and Abu-Ghannam [6] evaluated bound extracts of BSG and obtained 80% inhibition of DPPH, which is consistent with our results. This change coincides with the results obtained in Section 3.5, because the SSF process increased the concentration of free phenolic compounds, and therefore, high DPPH inhibition percentages were obtained. Dulf et al. [60] used the DPPH assay to evaluate antioxidant potentials in extracts of apricot byproducts subjected to the SSF process and reported an increase of 18% in the samples subjected to SSF.

## 4. Conclusions

The extrusion process, in combination with SSF for 48 h with *Fusarium oxysporum*, increased the release of soluble AX from BSG, obtaining yields 319 times more than those from BSG that were not subjected to any treatment. In the same way, the combination of both processes increased TPC and the proportion of free phenolic acids (ferulic acid, caffeic acid and *p*-coumaric acid) and increased the % inhibition of DPPH over values for BSG not subjected to any treatment. On the other hand, the results of this work show the potential use of both treatments to obtain secondary metabolites and hydrolytic enzymes, which hold high commercial value for potential use in the food and pharmaceutical industries. In addition, the combination of these two processes is of great interest to industries that desire to stop using chemical agents and to reduce processing costs.

## Figures and Tables

**Table 1:** Experimental design of the study.

Factor A Extrusion Process	Factor B Solid-State Fermentation
BSG non-extruded	BSG non-fermented
BSG extruded	BSG fermented

**Table 2:** Structural carbohydrates of brewers’ spent grain without treatment (BSG) and water and ethanol extracts.

Compound	g/100 g BSG Dry Basis ^1^
Cellulose	13.8 ± 0.1
Hemicellulose	26.4 ± 0.5
Lignin	18.9 ± 0.2
Water-soluble extract	10.9 ± 0.9
Ethanol-soluble extract	13.2 ± 0.3

^1^ Values are the average of triplicate ± standard deviation.

**Table 3:** Chemical composition of the unfermented BSG samples ^1^.

Compound	BSG Untreated (g/100 g Dry Basis)	BSG Milled (g/100 g Dry Basis)	BSG Extruded (g/100 g Dry Basis)
Moisture	2.2 ± 0.2 ^c^	4.2 ± 0.1 ^b^	8.4 ± 0.1 ^a^
Protein	21.6 ± 0.5 ^a^	22.2 ± 0.1 ^a^	21.8 ±0.3 ^a^
Lipids	11.9 ± 0.2 ^a^	11.7 ± 0.3 ^a^	12.0 ± 0.1 ^a^
Ash	4.6 ± 0.1 ^a^	4.7 ± 0.1 ^a^	4.0 ± 0.0 ^a^
Total carbohydrates	59.7	57.2	53.8
Total dietary fiber	61.8 ± 0.6	63.8 ± 0.5	62.9 ± 1.8
Insoluble fiber	61.6 ± 0.5 ^a^	61.8 ± 0.5 ^a^	60.7 ± 1.1 ^b^
Soluble fiber	0.2 ± 0.1 ^b^	2.0 ± 0.0 ^a^	2.2 ± 0.7 ^a^

^1^ Values are expressed as mean ± standard deviation (*n* = 3), except insoluble and soluble fiber values, which are the mean ± standard deviation (*n* = 2). The total carbohydrates are calculated by difference, and total dietary fiber is the sum of insoluble and soluble fibers. Values with different superscript letters in the same row are significantly different (*p* ≤ 0.05).

**Table 4:** Antioxidant capacity of free phenolic extracts from unfermented samples (brewers’ spent grain untreated (BSG), BSG milled (BSGm) and BSG extruded (BSGe)) and the fermented counterparts by *Fusarium oxysporum* after 48 h and 96 h ^1^.

Sample	Antioxidant Capacity of Free Phenolic Extracts Determined by the DPPH Assay	
	% Inhibition	DPPH Values (µmol TE/kg)
BSG	57.14 ^c^	1761.58 ± 55.67 ^c^
BSGm	67.74 ^a^	2207.00 ± 57.53 ^a^
BSGe	60.26 ^b^	1892.63 ± 51.63 ^b^
BSGf 48 h	93.17 ^a^	3276.05 ± 35.97 ^a^
BSGmf 48 h	92.9 ^a^	3265.28 ± 1.96 ^a^
BSGef 48 h	91.76 ^b^	3216.95 ± 31.53 ^b^
BSGf 96 h	92.56 ^a^	3250.76 ± 21.54 ^a^
BSGmf 96 h	92.96 ^a^	3267.26 ± 22.34 ^a^
BSGef 96 h	90.76 ^b^	3174.85 ± 23.94 ^b^

^1^ Values are averages of triplicate ± standard deviation. Values with different superscript letters in the same column are significantly different (*p* ≤ 0.05).

## Data Availability

The data presented in this study are available in article.

## Abbreviations

BSG, Brewers’ spent grain; BSGe, Brewers’ spent grain extruded; BSGm, Brewers’ spent grain milled; AX, Arabinoxylans; SSF, Solid-state fermentation; DPPH, 2,2-Diphenyl-1-picrylhydrazyl; TPC, Total phenolic compounds.
